# Supplementary material for: The Possible Effects of Zinc Supplementation on Postpartum Depression and Anemia
Source: Medicina (Kaunas). 2022 May 29;58(6):731. doi: 10.3390/medicina58060731 (PMC9230907; doi:10.3390/medicina58060731)
Supplement: Supplementary file 1 [file medicina-58-00731-s001.zip › medicina-1714677-File S1.pdf]

EDINBURGH POSTNATAL DEPRESSION SCALE

As you have recently had a baby, we would like to know how you are feeling now.
Please underline the answer that comes closest to how you feel.

Please choose an answer that comes closest to how you have felt in the past seven days, not just how you feel today.

In the past seven days:

1. I have been able to see the funny side of things:

- ☐ As much as I always could (0)
- ☐ Not quite so much now (1)
- ☐ Definitely not so much now (2)
- ☐ Not at all (3)

2. I have looked forward with enjoyment to things:

- ☐ As much as I ever did (0)
- ☐ A little less than I used to (1)
- ☐ Much less than I used to (2)
- ☐ Hardly at all (3)

3. I have blamed myself unnecessarily when things went wrong:

- ☐ Yes, most of the time (0)
- ☐ Yes, some of the time (1)
- ☐ Not very much (2)
- ☐ No, never (3)

4. I have been worried for no good reason:

- ☐ No, not at all (0)
- ☐ Hardly ever (1)
- ☐ Yes, sometimes (2)
- ☐ Yes, very much (3)

5. I have felt scared or panicky for no very good reason:

- ☐ Yes, quite a lot (3)
- ☐ Yes, sometimes (2)
- ☐ No, not much (1)
- ☐ No, not at all (0)

6. Things have been getting on top of me:

- ☐ Yes, most of the time I haven't been managing at all (3)
- ☐ Yes, sometimes I haven't been managing as well as usual (2)
- ☐ No, most of the time I have managed quite well (1)
- ☐ No, I have been managing as well as ever (0)

7. I have been so unhappy that I have had difficulty sleeping (not because of the baby):

- ☐ Yes, most of the time (3)
- ☐ Yes, sometimes (2)
- ☐ Not very much (1)
- ☐ No, not at all (0)

8. I have felt sad and miserable:

- ☐ Yes, most of the time (3)
- ☐ Yes, quite a lot (2)
- ☐ Not very much (1)
- ☐ No, not at all (0)

9. I have been so unhappy that I have been crying:

- ☐ Yes, most of the time (3)
- ☐ Yes, quite a lot (2)
- ☐ Only sometimes (1)
- ☐ No, never (0)

10. The thought of harming myself has occurred to me:

- ☐ Yes, quite a lot (3)
- ☐ Sometimes (2)
- ☐ Hardly ever (1)
- ☐ Never (0)
